# The Role of Artificial Intelligence in the Prediction, Diagnosis, and Management of Cardiovascular Diseases: A Narrative Review

**DOI:** 10.7759/cureus.81332

**Published:** 2025-03-28

**Authors:** Mohammed Farooque W Shaikh, Murtaza S Mama, Sri Harika Proddaturi, Juan Vidal, Pritika Gnanasekaran, Mekala S Kumar, Cleve J Clarke, Kalva S Reddy, Hasiya M Bello, Naama Raquib, Zoya Morani

**Affiliations:** 1 Internal Medicine, Dow University of Health Sciences, Dow International Medical College, Karachi, PAK; 2 Medicine, Medical University - Pleven, London, GBR; 3 Internal Medicine, Gandhi Medical College, Secunderabad, IND; 4 Medicine, Universidad del Azuay, Cuenca, ECU; 5 Emergency Medicine, Global Medical Center and Hospital, Salem, IND; 6 Internal Medicine, Sri Venkata Sai (SVS) Medical College, Hyderabad, IND; 7 College of Oral Health Sciences, University of Technology, Jamaica, Kingston, JAM; 8 Emergency, Buraydah Central Hospital, Buraydah, SAU; 9 Obstetrics and Gynecology, Grange University Hospital, Newport, GBR; 10 Family Medicine, Washington University of Health and Science, San Pedro, BLZ

**Keywords:** artificial intelligence in cardiology, cardiovascular disease diagnosis, cardiovascular diseases (cvds), machine learning in healthcare, personalized medicine

## Abstract

Cardiovascular diseases (CVDs) remain the leading global cause of mortality, and a high prevalence of cardiac conditions, including premature deaths, have increased from decades until today. However, early detection and management of these conditions are challenging, given their complexity, the scale of affected populations, the dynamic nature of the disease process, and the treatment approach. The transformative potential is being brought by Artificial Intelligence (AI), specifically machine learning (ML) and deep learning technologies, to analyze massive datasets, improve diagnostic accuracy, and optimize treatment strategy. The recent advancements in such AI-based frameworks as the personalization of decision-making support systems for customized medicine automated image assessments drastically increase the precision and efficiency of healthcare professionals. However, implementing AI is widely clogged with obstacles, including regulatory, privacy, and validation across populations. Additionally, despite the desire to incorporate AI into clinical routines, there is no shortage of concern about interoperability and clinician acceptance of the system. Despite these challenges, further research and development are essential for overcoming these hurdles. This review explores the use of AI in cardiovascular care, its limitations for current use, and future integration toward better patient outcomes.

## Introduction and background

Cardiovascular diseases (CVDs) have consistently been the leading cause of global mortality, accounting for approximately 30% of all deaths worldwide. Despite ongoing efforts to reduce their burden, the incidence of premature deaths related to CVD continues to rise, with an estimated 19.8 million deaths attributed to cardiovascular causes in 2022 [1]. Screening and managing CVDs at the global level remains a significant challenge due to the complexity of these conditions and the dynamic nature of their progression [2]. For comparison, cancer-related premature mortality was 72.9 per 100,000 in 2022, and respiratory diseases accounted for 42.1 per 100,000 [2]. Screening and managing them globally is challenging and requires efficient tools. Artificial Intelligence (AI), which has become an integral part of various fields, can be used to tackle this global burden. It provides the advantage of screening vast amounts of data in a fraction of the time while being cost-effective. It can be used without much expertise. Machine learning (ML), a subset of AI, uses different algorithms to recognize patterns in the data and predict the outcomes. Deep learning, which is a subset of ML, simulates the human brain using neural networks to perform complex tasks. AI has significantly contributed to medicine, particularly in a cardiovascular specialty, integrated with electrocardiogram (ECG), echocardiography, coronary computed tomography angiography (CCTA), and intravascular ultrasound, among other imaging techniques. AI can also be used for risk stratification, patient education, and medication adherence checks. For patient education, AI-driven chatbots guide patients in managing chronic conditions [3].

While numerous studies have focused on the application of AI in cardiology, many of these studies lack a thorough evaluation of the methodologies employed. For example, deep learning models applied to ECG analysis have demonstrated high diagnostic accuracy, but issues such as dataset bias and lack of transparency in algorithm decision-making remain unaddressed. Deep neural networks could outperform human cardiologists in detecting arrhythmias, with an F1 score of 0.837 and a receiver operating characteristic (ROC) curve of 0.97 [4]. However, these models often rely on datasets that lack diversity, which may limit their generalizability across different patient populations. Furthermore, the black-box nature of many AI algorithms poses significant challenges for clinical acceptance and integration. AI systems need to be transparent, with clearly defined decision-making processes that clinicians can understand and trust [5]. In another area, AI’s role in echocardiography and imaging has been extensively researched. For instance, Zhang et al. demonstrated that AI-enhanced echocardiograms could achieve diagnostic accuracy comparable to human experts in evaluating left ventricular ejection fraction (LVEF). However, these studies often fail to critically appraise the imaging protocols used across different institutions, which can affect the consistency and reliability of results [6].

When reviewing the studies on AI in cardiovascular care, it is essential to evaluate the methodologies employed. A study on using convolutional neural networks (CNNs) for CCTA highlighted that while AI models achieved high accuracy in predicting coronary artery stenosis, the study did not sufficiently address the challenges related to data quality and computational resources. AI-based models often require large, annotated datasets for training, which may not always be available, especially in resource-limited settings [7]. Moreover, biases in training data, such as overrepresenting certain demographic groups, could impact the model's performance in underserved populations. Thus, future studies must include more diverse datasets and provide detailed evaluations of model performance across various population groups to ensure generalizability and clinical applicability. Furthermore, while AI models demonstrate impressive diagnostic capabilities, the clinical validation of these systems remains a critical issue. Despite the promising results of AI in predicting conditions like atrial fibrillation and CAD, there is insufficient evidence to support the widespread clinical adoption of these technologies without rigorous validation through large-scale, multicenter trials [8].

In conclusion, integrating AI in cardiovascular care presents significant opportunities for improving diagnostic accuracy, personalizing treatment strategies, and enhancing patient monitoring. However, it is essential to critically evaluate the methodologies employed in AI studies, particularly regarding dataset diversity, algorithm decision-making transparency, and clinical validation. Future research should focus on overcoming the current limitations of AI in cardiology by ensuring that AI models are generalizable, interpretable, and rigorously validated across diverse patient populations. By addressing these issues, AI has the potential to revolutionize cardiovascular care and improve patient outcomes worldwide.

## Review

Use of AI in improving cardiology diagnosis

MI models can be evaluated through the assessment metrics, including F1 score and sensitivity and specificity. The F1 score provides a balanced measurement between precision and recall in addition to sensitivity and specificity, indicating correct positive and negative predictions [9]. AI techniques are frequently applied in cardiac imaging studies analyses [10]. Medical picture diagnostic processes at hospitals depend on CNNs as well as alternative ML models for interpreting ECGs, computed tomography (CT) scans, and magnetic resonance imaging of the heart [11]. Hannun et al. showed through research in 2019 that deep network models could outperform human cardiologists at detecting arrhythmias and ECG abnormalities. Deep neural networks proved superior to cardiologists in diagnosis, where F1 scores averaged 0.837 and ROC curve scores reached 0.97. The study outcome suggests AI and deep learning hold the potential for improving ECG analysis since they both produce more precise results with fewer automated ECG misdiagnoses and simultaneously help experts identify essential results by highlighting crucial information. The data demonstrates that AI works better with medical staff to enhance clinical diagnoses [12].

Using wearable ECG monitoring and AI, another research was able to predict the incidence of atrial fibrillation. The researchers detected Atrial fibrillation using CNNs and AI, an electrocardiographic characteristic, in conventional 10-second, 12-lead ECGs, even in benign sinus rhythms. The study comprised more than 180,000 people with verified rhythm classifications and ECGs collected between 1993 and 2017. The AI-enhanced ECG has a sensitivity of 79%, specificity of 79.5%, and accuracy of 79.4% in identifying atrial fibrillation; its area under the curve (AUC) is 0.87. It seems that AI might help identify people at risk for atrial fibrillation, even if their sinus rhythm is expected, which could lead to earlier diagnosis and treatment of the problem [13].

In cardiology, echocardiography is a standard diagnostic method for many heart conditions. A reliable diagnosis of cardiovascular illness requires a thorough study of left ventricular systolic function. The assessment of global longitudinal strain is particularly vulnerable to echocardiography mistakes caused by incompetent operators or differences in skill across researchers, even though the procedure is non-invasive and runs in real-time. An answer has emerged in the form of AI that can correctly recognize heart anatomy, determine ventricular capacity, and evaluate heart mechanics. Echocardiographic images may reveal particular valve disorders, and an AI system was developed in 2021 to help detect these abnormalities [14]. Furthermore, to assess cardiac function, LVEFs were automatically calculated using a deep-learning approach to echocardiography images [15].

**Figure 1:**
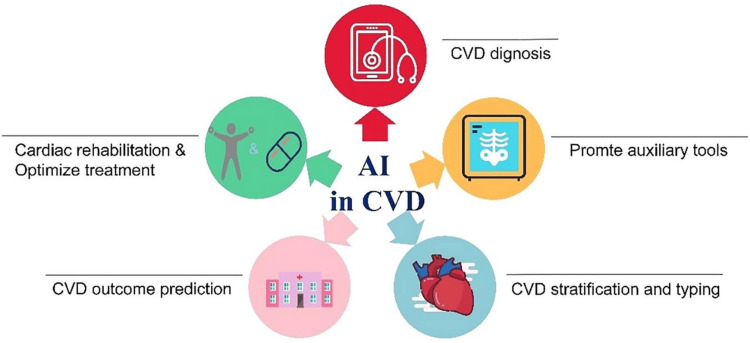
Role of artificial intelligence in cardiovascular diseases. Reproduced under CC BY 4.0. Publisher BMC [19].

The integration of AI in cardiology, particularly in echocardiography and multimodal cardiac imaging, faces several technical and clinical barriers. AI's most significant technical limitation is that we need large and diverse datasets to train AI models, but data privacy may pose problems, and may not have a lot of public data to train the AI model [16]. The intensely opaque characteristics of many AI algorithms create a barrier for clinicians to understand decision-making processes, thus leading to medical-legal concerns because clinical staff require absolute clarity about the algorithms' pathophysiologic mechanics. Different institutions need standardized protocols and algorithm validation to maintain reliable portable AI solutions in clinical settings [17]. Pediatric echocardiography diagnostic workflow benefits from federated learning and explainable AI, which successfully integrates to advance three diagnostic aspects of view recognition and disease classification together with cardiac function assessment [16]. As demonstrated by synergy-focused initiatives, the successful removal of barriers requires joint work by clinicians, scientists, and industrial stakeholders to connect AI systems with clinical expertise [18]. Developing robust AI applications in cardiology depends on collaboration between central hospitals and business institutions to enhance data diversity and algorithm generalization abilities [17]. The capacity to understand and physicians have faith in AI advice when used in conjunction with their clinical experience. Figure 1 shows the application of AI in CVD.

According to AI research, customizing cardiovascular medication based on individual patient profiles, including genetic characteristics, medical histories, and treatment responses, offers tremendous promise [20]. AI-based research has used ML to forecast statin responses and identify genetic and clinical variables [21]. Transcatheter aortic valve replacement (TAVR) is one procedure that can benefit from AI's ability to shed light on the valves' locations and size, which might shorten the procedure and reduce complications [10]. AI also offers procedural guidance for angiography and TAVR, including coronary angiography, and benefits imaging and decision-making. Applying the AI to the transformation of the given problem into an AI-driven system that models spatial coordinates to gain views, it thus proved 89% successful in the simulations [22] and improved imaging accuracy and efficiency in the intra-cardiac echocardiography (ICE). A human error reduction and TAVR procedural outcomes improvement with a high correlation to manual measurements by the expert is achieved by AI to automate anatomical measurements from CT scans [23]. AI algorithms also predict a better fluoroscopy angle for the procedure, beyond current trial and error fluoroscopy limitations, and increase procedural efficiency [24]. In addition to visualization, augmented reality systems have enhanced visualization by providing a 3D environment and real-time feedback that improves catheter positioning accuracy and provides training devices to interventionalists [25]. Collectively, these advancements come together to do a better job of improving the procedural guidance by optimizing the imaging angle and valve placements to get better clinical results.

These AI-powered technologies might revolutionize heart care by facilitating the delivery of precise and individualized treatments. Personalized treatment plans, more accurate procedures, fewer risks, and better patient outcomes are all within reach with the help of these AI-powered devices [14]. However, complete validation, improvement, and seamless integration into existing processes are necessary for AI technology incorporation in procedural systems. Priorities for future research in this area should include making AI algorithms more adaptable to different procedural situations, guaranteeing their accuracy, and encouraging frameworks for cooperation between healthcare professionals and AI. Redefining therapeutic paradigms and improving the quality of care for cardiology patients are specific outcomes of the effective use of personalized cardiovascular treatment entering a new era with AI in procedural guiding [26,27].

When it comes to the planning and administration of treatments for long-term heart problems, AI is crucial for improving personalized patient care. By analyzing vast datasets and intricate patient profiles, AI can create individualized treatment plans that consider differences in medical history, pharmacological reactions, and genetic predispositions [26,27]. AI allows more accurate treatment outcome prediction and personalized therapy using ML approaches. Furthermore, AI helps optimize procedure planning, minimize complications, and improve patient safety in interventional cardiology therapies. Healthcare providers may better manage chronic cardiac conditions using AI-driven decision support systems, which allow for more tailored cardiovascular care, more effective therapy, and better patient outcomes overall [28].

The use of AI in cardiology has many benefits, including the increased focus on the human element of Healthcare [29]. Healthcare providers rely on accurate recording of patient treatment in their medical records, and AI is a tool that shows promise in this area [30]. By using AI technology, healthcare providers have the potential to enhance the recording of vital patient data, including diagnoses, treatments, and other pertinent information [31]. AI-powered systems can automate data entry, categorize and encode medical records, and lend a hand in creating comprehensive electronic health records (EHRs) [32]. Also, by instantly spotting likely errors, conflicts, or missing data, AI has the potential to improve the accuracy and completeness of medical data. This enhances patient safety, reduces the likelihood of medical errors, and fortifies patient documentation [33]. In order to speed up the documentation process, healthcare practitioners could benefit from voice recognition and natural language processing technology driven by AI to help them translate verbal notes into written documentation [34].

In the realm of medical records, AI may not only increase efficiency but also simplify data processing and pattern detection. Clinicians may be able to use this information to spot patterns, possible danger signs, and individualized treatment plans. It is believed that using medical record recording utilizing AI would improve data accuracy, simplify administrative procedures, and give healthcare providers more time to concentrate on patient-centered, high-quality treatment [35]. While there are many benefits to using AI to capture medical records, doing so requires constant validation and improvement to guarantee the most excellent standards of accuracy, dependability, and regulatory compliance. Future efforts in this field should aim to improve the interpretive skills of AI algorithms, strengthen cybersecurity protocols to protect patient information, and promote smooth interaction with current healthcare systems. A more efficient, precise, and patient-centered healthcare system may be possible due to advancements in AI-driven documentation, which may completely alter administrative processes [36,37].

Cardiology administrative tasks might also be amenable to automation using AI and language models. Healthcare providers may have more time to focus on direct patient care and operational efficiency if these technologies automate routine administrative tasks [38]. These processes may be automated to reduce the likelihood of errors or inconsistencies in the patient record. These technological advancements make more time spent with patients and returning to medicine's personal touch possible. Incorporating AI into systems that monitor patients may have several further benefits. With the use of AI, vital signs detect issues early on, vital signs including heart rate, blood pressure, and respiration rate may be monitored in real-time, the administration of medicines promptly, and ultimately, better results [39]. Research conducted in 2019 by Hannun et al. showed that AI algorithms can identify arrhythmias and ischemia in the ECG data provided, leading to earlier diagnoses of atrial fibrillation [12]. Several devices can now monitor vital signs and provide real-time feedback, thanks to AI, which has improved patient monitoring in many settings [40]. A recent study introduced a novel approach to improve the interpretation of ECG data, specifically to enhance the accuracy of peak location identification and heart condition detection. This approach combines two event-related moving averages with fractional Fourier transform (FrFT) [41]. Using these devices to monitor vital signs and occurrences like malignant arrhythmias has tremendous potential in cardiology [42]. AI can potentially increase healthcare efficiency by evaluating massive datasets quickly and accurately, leading to less work for healthcare workers and better patient care [43].

In conclusion, wearable technology like trackers and smartwatches enables remote monitoring, which enhances healthcare outcomes by providing ongoing insights into the patient's health [44]. The AI-powered wearable devices provide the further capability to monitor vital signs and conditions like malignant arrhythmias with simultaneous real-time feedback and early intervention [40]. A new approach of two events-related moving averages with the help of FrFT was introduced to enhance peak detection accuracy in ECGs. It was shown that this technique would also enhance heart condition detection and enable the ECG data to be read better [41]. The advances allow us to see patient monitoring and safety's vital role in offloading work to HC professionals and earlier interventions [45].

Heart Failure

​Heart failure (HF) is a significant complication of hypertension and contributes notably to hospitalizations in China. A study involving 13,678 HF patients from 132 hospitals from January 2012 to September 2015 reported an in-hospital mortality rate of 4.1%. Additionally, research indicates that HF accounts for a considerable portion of inpatient care, highlighting its impact on healthcare resources [46, 47]. XGBoost (Washington, DC), Random Forest (Bhopal, India), and Support Vector Machine (Bells Lab, Murray Hill, NJ) have all been shown to have great prediction and diagnosing potential for left ventricular diastolic dysfunction (LVDD) and left ventricular hypertrophy (LVH). Most ECG indices based on voltage have low sensitivity (19%-25%); these models perform much better. An improvement in sensitivity for detecting LVH with AI integration from 42% to 69% is accompanied by a small decrease in specificity (from 92%-94% to 87%) [48]. This tradeoff means more early disease detection but more likely false positives that give follow-up evaluation. In real-world scenarios, this trade-off should be well managed to avert overburdening of the healthcare systems while maximizing the auxiliary benefit of early detection.

First, it is vital that the population characteristics and the dataset size impact model generalizability. For instance, an ML-based study of whether LVDD can be detected using ECGs in a multicenter cohort laboratory study report showed excellent diagnostic accuracy (AUC = 0.83, sensitivity = 78%, specificity = 77%) [49]. However, such biases in dataset diversity may limit the model’s performance in patient populations. It is important to ensure diversification and representativeness of datasets during training in order to make such applications more general. Using radionics to extract quantitative features from imagery data has proved helpful in the differentiation of HCM versus hypertension. It achieved an accuracy of 85.5% compared to the accuracies that one would achieve with the manual expert interpretation, which, typically, is at about 64% accuracy [50]. Yet, radiomics has limitations such as overfitting problems, notably when trained with small datasets, and difficulties interpreting practical clinical relevancies. The remaining key challenges are bias in training datasets and lack of algorithm validation across diverse populations. Conversely, a model trained from a specific demographic may perform poorly on more underserved groups and, therefore, be less generalizable. Also, many AI algorithms are black boxes, so clinicians do not trust or understand their predictions. One way to solve this problem would be to incorporate explainable AI techniques to offer a transparent and interpretable decision-making process.

Despite its progress, AI use in ECG analysis also has clinical concerns. This would enhance sensitivity as the AI may be able to pick up on early or subtle abnormalities that traditional methods would miss. However, reduced specificity may cause overdiagnosis and treatments without need. In medical practice infants, the tradeoff between sensitivity and specificity must be consistent with the prevalence of the disease and the risks of false positive and false negative diagnosis. Introducing this new risk factor for conditions like HF using AI for predictive modeling has brought in endothelin-1, a factor linked to oxidative stress and cardiac restructuring [47]. However, more research is needed regarding the availability of randomized trials to validate the use of AI-based therapeutic strategies to translate predictions into care improvements.

For the diagnosis of HF, the ECG has been utilized for many years for the screening of cardiac disease, but in the context of LVH screening, traditionally, the voltage index, as Sokolow-Lyon and Cornell, has inferior sensitivity, making them a weak tool for screening, for that reason, a meta-analysis about the uses of AI with ECG found that it uses potentiate the sensitivity from 19-25% of the traditional methods to 69%, but with reducing specificity from 92-94% to 87%, with better diagnostic accuracy for detection of LVH [48]. On the other hand, multiple models using ML have been proposed; one study proposed using ML with 12 lead electrocardiography input in a CNN to diagnose LVDD in the context of dyspnea; this model seems to outperform the use of NT-proBNP to discriminate pulmonary from cardiac dyspnea. Another promising tool is the ML used to evaluate LVDD on ECG compared to echocardiography, a multicenter prospective study quantifies left ventricular relaxation velocities (e´), and they obtained a good correlation with the measured by ECG, AUC 0.83, sensitivity 78%, specificity 77%, negative predictive value 73% and positive predictive value (PPV) 82%, in the internal test, and a similar ability in the external test [49]. The other image techniques for the diagnosis of structural heart disease have been the objective of the study in the AI era; for example, with the use of Radiomics, which obtains multiple image characteristics using an algorithm, it showed to correctly differentiate HCM from hypertensive heart disease with an accuracy of 85.5%, which is better than the current method, 64% [51,52].

Coronary Artery Disease

Sensitivity is often fostered using the Jaccard index and dice coefficient, which are widely used metrics to evaluate image segmentation algorithms in medical imaging. The Jaccard Index is the size of the intersection divided by the size of the union of the sets of samples [53]. Just as the Dice Coefficient calculates how much overlap between two samples, it is also the case for the Dice Coefficient, which computes twice the intersection of the two sets and divides by the sum of the sizes of the two sets. These metrics are important for assessing AI models' accuracy in delineating such regions of interest as coronary artery lesions [54].

Of all AIs, those for Fractional Flow Reserve using CT (FFR-CT) in CAD evaluation have shown promising diagnostic accuracy in the context of CAD evaluation. Lipkin et al. compared CCTA with AI quantitative CT (AI-QCT) interpretation to myocardial perfusion imaging (MPI) in terms of detecting obstructive CAD. AI-QCT had a higher AUC than MPI (0.88 vs 0.66) for predicting > 50% stenosis in the 301 patients from the CREDENCE trial [55]. Chiou et al. also compared AI-QCTISCHEMIA to CT-FFR and physician visual interpretation in 442 patients. Results from AI-QCTISCHEMIA were better (specificity and DUrugia) than clinician interpretation (specificity of 0.62) and CT-FFR (specificity of 0.76) or DUrugia (specificity of 0.91) [56]. On the other hand, the challenges are computational demands and the need for large, annotated datasets. According to Pershina et al., the diagnostic accuracy of FFR-CT (AUC = 0.90) depends heavily on the data quality and computational resources [57]. Population homogeneity biases arise in studies. As Martin et al. mentioned, AI-based CT-FFR studies exclude high-risk patients [58]. That limits generalizability. Accuracy vs. interpretability tradeoffs is crucial. While the DenseNet201 models are highly accurate, they have a black-box nature that makes it challenging to trust in the clinical setting. While DenseNet121 performed better than thoracic radiologists, the absence of transparency from the model could delay clinical adoption by Sufian et al. [59].

For coronary artery stenosis, the use of CCTA is key for CAD, with an excellent negative predictive value of 99%, the use of CNN models can potentiate its utility, as shown in a study which aimed to diagnose and classify the severity of the coronary stenosis with an accuracy of 0.77 (sensitivity) and 0.80 (specificity), respectively. Besides, CCTA aided with ML algorithms can be a good alternative to invasive FFR, the gold standard, to evaluate functional stenosis; as shown in a study, the utilization of AI algorithms in FFR-CT can predict accurately the function stenosis when compared with the gold standard, with an accuracy of 83.2%, an excellent correlation (r=0.9994) [51]. Using CCTA has provided valuable data for developing a predictive model incorporating 44 CCTA features and 25 clinical factors using ML. This model demonstrated superior performance in predicting five-year all-cause mortality, with an AUC of 0.79, significantly outperforming the Framingham Risk Score, which had an AUC of 0.61 [51].

**Figure 2:**
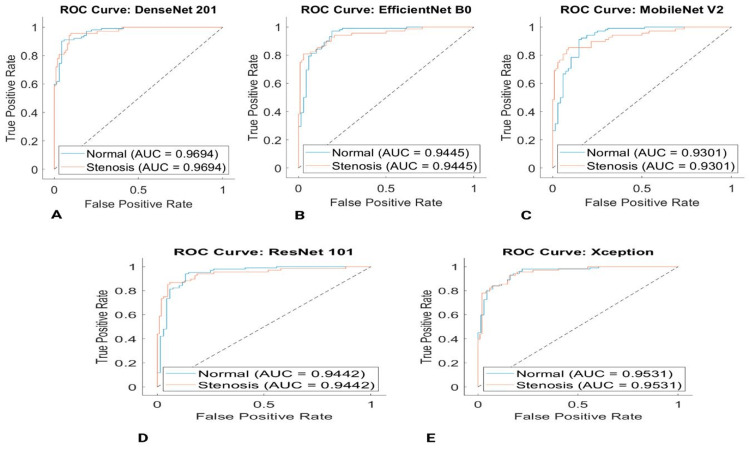
The various types and patterns of ROC curves comparing the true positive and false positive rates for coronary artery classification are shown in five different images: (A) DenseNet201, (B) EfficientNet-B0, (C) MobileNet-v2, (D) ResNet101, and (E) Xception. Reproduced under CC BY 4.0. Publisher MDPI [60].

EfficientNet-B0, DenseNet201, ResNet101, Xception, and Mobilenet-v2 models are suggested for automated coronary artery segmentation and classification by Kaba et al. (Figures 2A-2E). DenseNet201 outperformed other pre-trained classification models, achieving an impressive accuracy of 0.90, a specificity of 0.9833, a PPV of 0.9556, a Cohen's Kappa of 0.7746, and an AUC of 0.9694, highlighting its superior performance in classification tasks [60].

Myocardial Infarction

Studies using lipid profiles to evaluate cardiovascular risk levels found strong statistical associations through p-values less than 0.001 between patient phenogroups. The research by Xue et al. used unsupervised ML to create distinct phenogroups of ST-segment elevation myocardial infarction (STEMI) patients through their lipid expression patterns. ANOVA and Cox proportional hazards models from the research demonstrated that the studied groups displayed noteworthy differences in lipoprotein(a), High-Density Lipoprotein Cholesterol (HDL-C), and apolipoprotein A1 (ApoA1) patterns together with substantive clinical event prediction indications [61].

Further research should combine lipidomic information with genomic and proteomic methods to enhance phylogroup classification methods. Research following subjects over time would best confirm lipid-based phylogroup predictive ability through long-term assessments. Targeted therapies during clinical trials within phenogroups will generate innovative treatment methods for high-risk patients and improve their therapeutic outcomes [62].

These study findings can transform how healthcare professionals evaluate cardiovascular risks in their patients. Risk assessment tools that utilize phenogroups developed via ML enhance existing cardiovascular prediction models, while patient-specific lipid management according to phenotypic traits leads to improved medical procedures and treatment success. The process of phenotyping through unsupervised ML encounters specific implementation obstacles. The overfitting problem occurs mainly in small-sample dataset scenarios because models will detect noise patterns instead of meaningful clinical patterns. The clinical use of ML-derived clusters becomes complicated because it is difficult to determine the biological bases for grouping when cluster patterns lack clear explanatory logic. Different algorithm choices or parameter settings create problems because they result in inconsistent research results [63].

Resolving existing challenges demands strict model validation, combining biological information in data-science methods, and methodological transparency. The full realization of lipidomics and ML applications in patient-specific cardiac care demands these important advancements.

ST-segment elevation myocardial infarction is one of the manifestations of CAD, and it is associated with a risk of 5-6% mortality during hospitalization and up to 18% mortality risk within one year after the event; for this reason, a study conducted to classify STEMI patients based on their lipid profile using unsupervised ML, and they found three groups, the lipid profiles of the three groups were significantly different (p<0.001), with group 1 consisting of patients with the lowest ApoA1 and HDL-C levels as well as moderate levels of triglycerides (TG), total cholesterol (TC), and LDL-C. Group 2 was composed of patients with lower TC, TG, low-density lipoprotein cholesterol (LDL-C), and apolipoprotein B (ApoB), and group 3 had the highest apolipoprotein A1 (ApoA1) and high-density lipoprotein cholesterol (HDL-C) levels as well as intermediate levels of apolipoprotein B (ApoB), high-density lipoprotein cholesterol (HDL-C), and apolipoprotein A1 (ApoA1). Patients in group 1 had substantially greater odds of all-cause death, all-cause rehospitalization, and cardiac rehospitalization, the three primary outcomes. This study helps classify patients based on their lipid profile and stratified risks, but future studies are needed to help manage in a personalized manner these phenogroups of patients [64]. 

Echocardiography

**Figure 3:**
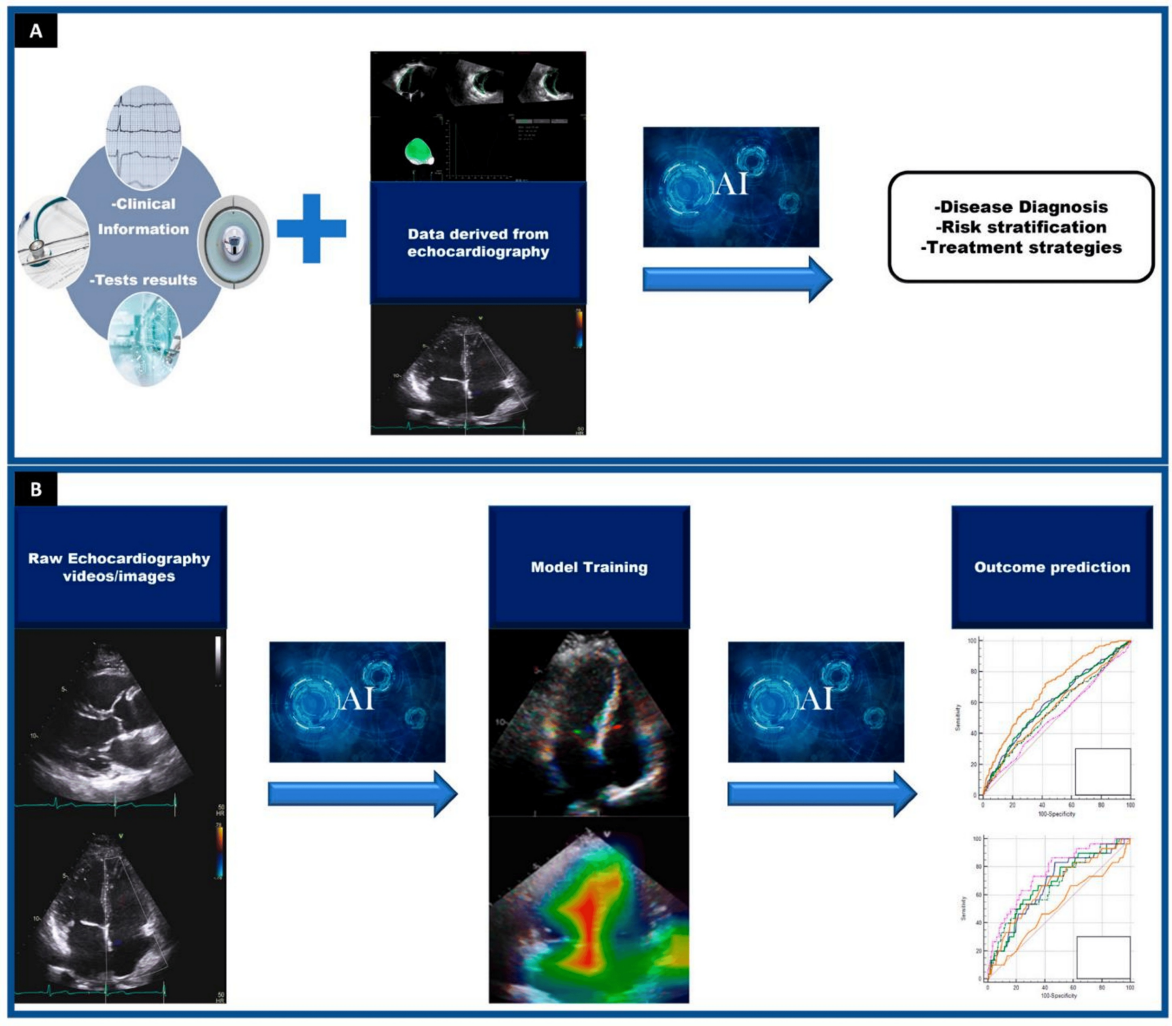
(A, B) Echocardiography AI applications Reproduced under CC BY 4.0. Publisher MDPI [65].

Echocardiography is vital for heart pathology diagnosis and treatment. One of the few real-time imaging methods that may identify problems is echocardiography. Accurate cardiac structure and function assessments aid clinical diagnosis and therapy selection (Figures 3A, 3B) [65]. The computerized assessment of left ventricular volume and function was the first use of AI in echocardiography. With AI, image quality, shorter scan times, and quicker segmentation, processing, and analysis may be enhanced. The program that Knackstedt et al. used to measure the ejection fraction of 255 patients and longitudinal strain from biplane images of the left ventricle is vendor-independent and employs a machine-learning approach [66]. Measuring EF and LS was successful in 98% of trials, taking an average of 8 ± 1 seconds per patient.

In comparison to the manually traced reference, the accuracy percentage was 92.1%. The use of speckle-tracking echocardiographic data in an ML framework was assessed for diagnostic utility by Narula et al. a year later to automatically distinguish between normal hypertrophy in athletes and HCM [67]. The specificity for distinguishing HCM from physiological hypertrophy was 82%, while the sensitivity for 77 athletes and 62 individuals with HCM, respectively, was 87%.

Using 14,035 ECGs acquired over a decade, Zhang et al. trained CNN models for several applications, such as automated viewpoint identification, cardiac chamber segmentation across five standard views, chamber volume quantification, left ventricular mass, LVEF, and longitudinal strain assessment automatically via speckle tracking [68]. When identifying mild, moderate, and severe mitral regurgitation, the corresponding detection accuracies were 99.52%, 99.38%, 99.31%, and 99.59%, respectively. The data for the study were compiled from 530,871 deidentified ECGs obtained from 171,571 men and 158,404 women. The median follow-up period was 4.1 years. The purpose of the data was to build an AI system that could determine the degree of aortic stenosis [69]. While the continuity equation correctly recognized 73.9% of patients with traditional high gradient aortic stenosis, the AI identified 95.3% of individuals. Aortic stenosis severity was established by the complete phenotypic Evaluation, which did not rely on the size or velocity of the left ventricular outflow tract. The technique has shown similar efficacy in severe aortic stenosis defined by low gradient and flow and in healthy and impaired left ventricular systolic function.

Recent research used AI-enhanced echocardiography measures to improve disease severity classification and identify high-risk subgroups in a trial that included over 2,000 patients with aortic stenosis [70]. It is possible that the timing of aortic valve replacements might be improved if this research identified higher-risk patients. These patients exhibited increased biomarker levels, a rise in late gadolinium enhancement, an increase in aortic valve calcium scores, and an increase in cases with unsatisfactory clinical outcomes [70]. ML for automated mitral and aortic disease screening of echocardiographic films was presented employing a validated cohort of 311 participants and an extensive training cohort of 1,335, as reported in a recent study [71]. To categorize echocardiographic images, detect valve heart sickness, and measure disease severity, our deep learning system achieved a high degree of accuracy (AOC > 0.88 for all left heart valve diseases) [71]. These latest findings confirm that an automated system can screen, quantify, and classify the severity of common medical conditions using standard echocardiographic datasets for training.

AI in cardiac and coronary CT scans

Mobile devices are increasingly integrated into echocardiography workflows, particularly for opportunistic risk categorization and remote monitoring. The study by Oikonomou et al. introduced the Digital Aortic Stenosis Severity Index (DASSi), an AI-based biomarker derived from echocardiographic and cardiac magnetic resonance (CMR) imaging. This tool enables opportunistic risk stratification, even through handheld devices, facilitating personalized screening and follow-up without the need for complex imaging setups [72]. While studies like Narula et al. [67] provide comprehensive statistical analyses, others, such as those focusing on AI-enhanced screening for mitral and aortic diseases, often lack methodological depth. For instance, Popat et al. conducted a meta-analysis evaluating AI algorithms for aortic stenosis (AS) screening, reporting a pooled sensitivity of 0.83 and specificity of 0.81, with an AUC of 0.909, indicating outstanding diagnostic accuracy. Metrics such as an AUC > 0.88, observed in AI models for left heart valve diseases, reflect superior discriminative performance compared to traditional diagnostic methods. For example, Tseng et al. highlighted that AI models trained on extreme-spectrum cohorts achieved an AUC of 0.91 for detecting severe AS, outperforming conventional ECG interpretations [73]. Despite promising results, AI in echocardiography has limitations. Potential biases in training datasets can affect model generalizability, as highlighted by spectrum bias issues in Tseng et al.'s study. Moreover, the interpretability of AI models remains a challenge, with “black-box” algorithms often lacking transparency. Clinical validation across diverse populations is essential to ensure robustness.

Ensuring consistency in echocardiographic data acquisition is critical, as variations in imaging quality can impact AI performance. Han et al. emphasized the need for standardized protocols to enhance model reliability in real-world settings [74]. Coronary artery calcium scoring is a novel approach to risk assessment and coronary atherosclerosis detection. On non-contrast ECG-gated coronary CT images, the Agatston scoring method may be used to assess the amount of coronary artery calcium [75]. Image noise, motion artifacts, or blooming artifacts caused by highly calcified arteries or devices might degrade the picture quality. In addition, time-efficient AI systems should focus on the CAC scoring technique since it is often labor-intensive. A supervised learning-based approach was proposed by Wolterink et al. to detect and quantify calcification in the coronary arteries in CCTA images without pre-tree extraction [76]. As part of this study, two hundred and fifty people were scanned in a row for cardiac CT exams. With a sensitivity level of 72%, the proposed method was designed to assess automated CAC in CCTA.

 In a recent study for CACS using non-contrast CT images, Martin et al. assessed Automated CaScoring, a research program developed by Siemens Healthineers (Erlangen, Germany) based on deep learning [77]. Under this approach, a CNN was used, which had been trained using 2,000 labeled datasets. The ML system had a 93.2% success rate compared to the human assessors when classifying 476 out of 511 patients into the same risk group. The Evaluation of coronary artery calcifications was carried out using ML algorithms using non-contrast low-dose chest CT data acquired during lung cancer screening. Agatston scores were directly extracted from 5,973 non-contrast, non-ECG gated chest CT data using a deep CNN [78]. The algorithm demonstrated remarkable performance with a Pearson correlation value of 0.93 and an accuracy rate of 73% for risk category classification. When it comes to detecting individuals with stress-induced ischemia, Van Hamersvelt et al. investigated the additional advantage of deep learning analysis of the left ventricular myocardium in coronary CT angiography to assess coronary stenosis severity [79]. This study's AI techniques included a multiscale CNN for automated LV myocardium separation. Afterward, all CT slices of the left ventricular myocardium were convolutionally auto-encoded to unsupervised characterize the approach. In the end, using a support vector machine, the obtained characteristics were used to classify individuals as having or not having functionally considerable coronary artery stenosis. Comparing the proposed method to classification relying just on the Evaluation of coronary stenosis degree yielded better discrimination (AUC = 0.76). The proposed method has an 84.6% sensitivity and a 48.4% specificity.

The NeXt sTeps experiment compared invasive coronary angiography with CT angiography for heart flow investigation in 254 patients, with FFR as the gold standard [80]. Our goal was to determine how different variables, such as coronary stenosis severity, FFRCT (derived FFR), and FFR (lesion-specific ischemia), relate. An analysis of plaque characteristics showed better separation of lesion-specific ischemia from stenosis alone. In a separate study, the same researchers looked at the possibility of using ML with quantitative stenosis, plaque metrics, and clinical data obtained from CCT to make accurate predictions of lesion-specific ischemia as measured by FFR [81]. Together, they improved the ability to foretell lesion-specific ischemia. Automatic detection, Evaluation, and classification of calcified and non-calcified plaque-related coronary stenosis were achieved by Kelm et al. using an ML approach [82]. Following centerline extraction and lumen segmentation, 229 CTA volumes were used to train their random forest model. In each case, the model accurately diagnosed stenosis and calculated the area of the lumen's cross-section in around 1.8 seconds on average. By training a recurrent CNN, Zreik et al. were able to accurately determine its composition, classify obstructive and non-obstructive coronary stenosis and detect coronary plaque, [83]. The approach has a 0.77 accuracy rate for detecting and describing coronary plaque. Our stenosis detection and anatomical significance evaluation accuracy was 0.80 using this approach.

AI in cardiac MRI

Before moving on with processing or analysis, AI may check that all captured photos meet a predetermined criterion. Some examples of what researchers have proposed for automated MRI tasks include determining aorta position, whether it is ascending or descending [84], identifying likely minimizing interslice motion artifacts while offering comprehensive coverage of the heart [30,31], and identifying basal and apical slices that may not be present [85]. An aspect of analyzing cardiac MRI scans is determining the ejection fraction, which may take up to twenty minutes for a trained cardiologist. Rapid image interpretation, with less inter- and intra-observer variability, will be much improved by an entirely automated approach [86,87]. In their work on LV detection using CNN, Xue et al. presented a reliable method. In order to train the CNN models, a total of 25,027 scans were collected. With a success percentage of 99.98%, the three-class model correctly recognized the left ventricle in 99.98% of the test situations [88]. Tan et al. created a neural network regression approach for automated left ventricle segmentation in cardiac MRI using short-axis and long-axis images. Throughout the whole cardiac cycle, this technique provides complete coverage from top to bottom [89]. The initial step was training a network to detect the myocardium and its hollow core. A network was then constructed to determine radii from the cavity center, allowing smooth endocardial and epicardial contours to be acquired. Du et al. generated ventricle outlines in short-axis pictures without pixel categorization but by using boundary regression on the two ventricles [90]. Bernard et al. also presented an automatic segmentation method that outperformed manual tracing with Dice similarity values of 0.95 or better [91]. An approach was presented by Fahmy et al. that uses deep CNNs to assess scar volume autonomously and left ventricular mass in patients with HCM (utilizing late gadolinium enhancement [92]. Rising interest in the field of radionics has been seen as of late. Radiomics is the process of extracting many quantitative imaging features from digital medical images using various statistical and mathematical methods to create high-dimensional data that can be analyzed [93]. Using radiomics on T1 and T2 mapping, it may be possible to distinguish between individuals with HCM and hypertensive cardiac disease [50].

Auxiliary tools are more effective by AI

AI techniques can potentially improve the performance of ancillary imaging tools, including CT, MRI, and echocardiography. LVEF is an essential metric when predicting the success of HF treatments like resynchronization and defibrillator implantation. One of the most common and accurate ways to measure ejection fraction in clinical practice is using two-dimensional echocardiography. Auto EF, developed from AI, is the model that can effectively calculate EF. In order to automate and accurately measure LVEF, Asch et al. presented an ML method that can match the accuracy of human clinicians in LVEF extraction [94]. This advanced algorithm improved clinical safety by allowing nurses to track patients' LVEF in real-time and eliminating the requirement for a trained sonographer. Furthermore, by analyzing ECGs, AI has enabled the monitoring of regional wall motion issues. Utilizing a matched dataset of patients with and without myocardial infarction, Kusunose et al. built a deep CNN to anticipate anomalies in wall motion [95].

One of the main ways to diagnose coronary artery stenosis is using CCTA, a non-invasive technique that is very successful. It requires semi-automated manual Evaluation and is costly, labor-intensive, and time-consuming. Compared to the level 3 expert reader consensus, Choi et al. showed that an AI-based technique improved the CCTA efficiency by facilitating the quick and precise assessment of atherosclerosis, stenosis, and vascular morphology [96]. In order to predict stenosis and plaque using coronary CT angiography, Lin et al. created a deep-learning model. When diagnosing myocardial infarction, our deep learning method could help predict the outcome [97]. In addition, a group of researchers examined the efficacy of AI algorithms in estimating myocardial blood flow (MBF) and myocardial perfusion reserve using CMR scans [98]. The study included 1049 patients and had a median follow-up of 605 days. Using AI to quantify CMR, the Cox proportional hazard model results showed that reduced myocardial blood flow and myocardial perfusion reserve are independent predictors of cardiac prognosis. Angiography is still relied upon for more than 70% of clinical treatment decisions, even though invasive FFR reserve is the best way to detect coronary stenosis.

Nevertheless, when FFR is less than 0.80, angiography does not accurately predict the presence of stenosis. As Hae et al. found, the visual-functional discrepancy between FFR and angiography may be reconciled with ML [99]. In addition, an ML model used extreme gradient boosting by Cho et al. to accurately forecast samples with FFR ≤ 0.80 and an AUC of 0.84 [100].AI models like auto EF and CNNs have demonstrated notable improvements over traditional methods. For example, Papadopoulou et al. showed that automated LVEF calculation using AI-enabled handheld ultrasound devices had a strong correlation with standard echocardiographic measurements (r = 0.90 for cardiologists and r = 0.84 for nurses), with sensitivity and specificity rates exceeding 90% [101]. This contrasts with manual EF calculations, which are time-consuming and subject to operator variability. In coronary CCTA, Han et al. demonstrated that AI-enhanced CCTA had a diagnostic accuracy (AUC = 0.870) superior to traditional methods (AUC = 0.781), significantly reducing diagnostic time and improving workflow efficiency [102].AI improves clinical workflows by automating image interpretation and risk stratification. Real-time LVEF tracking, as shown in Papadopoulou et al.’s study, allows nurses to identify cardiac dysfunction promptly, expediting interventions and potentially reducing hospitalizations. This real-time monitoring significantly enhances patient outcomes compared to periodic assessments in traditional practice. The cost and time savings from AI integration are substantial. AI models can process imaging data in minutes compared to hours for manual evaluations. This reduces the workload for clinicians, allowing more time for patient interaction and complex decision-making, ultimately improving patient care.

AI models outperform traditional biomarkers in predicting MI. Makimoto et al. demonstrated that a CNN model had an F1 score of 83% and an accuracy of 81% in MI recognition from ECGs, significantly outperforming physicians (F1 = 70%, accuracy = 67%). Furthermore, Ramirez et al. showed that explainable AI using vector cardiograms (VCGs) achieved an accuracy of 91.4%, highlighting the potential of AI to enhance diagnostic precision [103].AI reconciles discrepancies between FFR and angiography by integrating physiological and anatomical data. Chandana highlighted how CNNs can improve diagnostic accuracy in coronary assessments, reducing false negatives common in traditional angiography [104].AI enhances prognosis prediction by integrating multiple diagnostic factors. Scannell et al. developed AI-AIF, which improves MBF quantification in cardiac MRI, aiding in comprehensive risk prediction [105]. AI-driven cardiology tools offer superior accuracy, efficiency, and cost-effectiveness compared to traditional methods. However, ensuring model transparency, reducing data bias, and conducting extensive clinical validation are critical steps toward widespread adoption.

CVD stratification and type with the help of AI

By improving the accuracy of stratification and classification for patients with CVD, new AI-based approaches have the potential to overcome some of the limitations of current methods and optimize tailored treatment. Traditional approaches have failed to identify many patients who will respond to cardiac resynchronization therapy, therefore improved phenogrouping could help narrow the pool of candidates. Based on shared characteristics in baseline deformation measurements, left ventricular volume, and clinical data, Cikes et al. used an unsupervised ML approach to divide individuals into four groups [106]. Two of the four phenogroups associated with CRT with a defibrillator had a far better therapeutic impact than previously indicated when compared to an implanted cardioverter defibrillator. Similarly, despite medication breakthroughs, treatments were limited in patients with HF and a lower LVEF. On aggregated individual patient data from nine β blocker trials that were randomized, placebo-controlled, and conducted with neural networks, Karwath et al. used variational autoencoders and hierarchical clustering [107]. Both sinus rhythm patients and those with concomitant atrial fibrillation could have their predicted responses to β-blockers distinguished using the AI-driven clustering strategy.

The complicated etiology of atrial fibrillation is a cardiovascular condition. According to epidemiological studies, the probability of getting atrial fibrillation is increased when several risk factors are present [108]. Three separate clusters were identified by Proietti et al. using hierarchical cluster analysis using the EORP-AF General Long-Term Registry [109]. Cluster 3 performed worse than Cluster 1 and Cluster 2 during an average follow-up of 22.5 months regarding composite outcome, cardiovascular events, and all-cause mortality (which includes both metrics). This suggests that cluster analysis could help understand the clinical phenotypes and prognostic outcomes of patients with atrial fibrillation. It is possible to lessen the severity of the repercussions and provide more accurate evaluations of coronary function by reducing aortic pressure during coronary angiography. Training a one-dimensional CNN using two existing datasets from patients undergoing invasive coronary angiography at four European cardiac centers allowed Howard et al. to reliably detect real-time recordings of damped arterial waveforms [110]. The model can distinguish between normal, damping, and artifactual beats with a success rate of 99.4% when tested against evaluations performed in the internal core laboratory and a success rate of 98.7% when tested against an external core laboratory that did not participate in neural network training.

Individuals with hypertension have substantial phenotypic variability in terms of cardiovascular outcomes, comorbidities, and antihypertensive drug response. Therefore, it is of the utmost need to assign clinically relevant labels to individuals with hypertension, enhance treatment strategies, and predict prognostic outcomes. The Systolic Blood Pressure Intervention T trial participants' baseline parameters were used by Yang et al. to conduct an unsupervised, data-driven cluster analysis. The results showed four reproducible clusters, which were important for predicting cardiovascular events and treatment responses [111]. The primary CVD result was more likely to be experienced by Cluster 4. Cluster 2 has a higher potential for major adverse consequences, whereas only clusters 4 and 1 demonstrated effectiveness with intensive antihypertensive medication. Endocrine hypertension is another kind of hypertension that is either ignored or wrongly categorized as primary hypertension in modern treatment settings.

In order to differentiate PHT from other subtypes of EHT, Reel et al. used a supervised ML system to analyze plasma miRNAs, urine steroid metabolites, plasma steroids, tiny plasma metabolites, and plasma catechol O-methylated metabolites [112]. The random forest model outperformed the other five ML models in distinguishing between primary hypertension, Cushing's syndrome, pheochromocytoma/catecholamine-producing paraganglioma, and primary aldosteronism, achieving an AUC of 0.95 using 57 MOmics characteristics. Classifying EHT (PA + PPGL + CS) from PHT using 37 MOmics features, the simple logistic classifier achieved an AUC of 0.96. In the genes that code for sarcomere proteins, more than 1,400 mutations have been found in up to 60% of persons with HCM [113,114]. One way to make genetic testing more cost-effective is to find patients likely to have favorable HCM genotypes. To explore the possibility of using CMR to indicate HCM genotype status, Zhou et al. created a deep learning method based on nonenhanced cine CMR images [114]. An AUC of 0.80 and a precision of 78.43% were the results of 10-fold cross-validation, which assessed the model's performance on the internal dataset. The diagnostic performance was much better when the deep learning model was integrated with the Toronto score; this is 84.31% accurate and has an AUC of 0.84 than when the score was used alone.

Many models are trained on specific datasets that may not represent diverse populations, leading to potential biases. For instance, Liang et al. demonstrated that ML models for predicting genotype positivity in HCM performed exceptionally well (AUC = 0.92) on their dataset but may face challenges when generalized to other demographics [115]. AI models, especially those with complex architectures like deep learning networks, risk overfitting when trained on small or homogeneous datasets. High computational demands limit the feasibility of AI in resource-constrained healthcare settings. Chen et al. showed that AI models using ECG data (AUC = 0.89) outperformed traditional scoring systems, potentially reducing unnecessary genetic tests [116]. AI can identify high-risk individuals, streamline genetic counseling, and improve resource allocation. Traditional clinical risk scores, like the Toronto score, typically achieve AUCs around 0.70-0.75. AI models exceeding 0.80 demonstrate superior predictive accuracy. Morita et al. reported an AUC of 0.86 for an AI model predicting favorable genotypes in HCM, significantly outperforming the Toronto score (AUC = 0.75) [117]. Ishikita et al. highlighted that AI models predicting major adverse cardiovascular events (MACE) in Tetralogy of Fallot patients (AUC = 0.81) performed well in controlled datasets but required validation in diverse populations [118].AI enables risk stratification and treatment personalization based on genetic, imaging, and clinical data. Automated diagnostics and risk prediction models, like the AI-CAC (AUC = 0.816), improve workflow efficiency and reduce costs [119]. In conclusion, while AI offers significant advancements in CVD management, addressing limitations related to data quality, generalizability, and interpretability remains crucial for its widespread clinical adoption.

Discussion

AI has demonstrated significant potential in transforming the cardiology field by improving diagnostic accuracy, enhancing personalized treatment strategies, and facilitating the early detection of diseases. One of the key strengths of AI is its ability to analyze large and complex datasets quickly and accurately. Deep learning (DL) algorithms, especially CNNs, have shown exceptional performance in interpreting ECGs, detecting arrhythmias, and identifying heart disease markers that may go unnoticed in traditional manual interpretations [120]. For instance, AI applications have revolutionized cardiovascular imaging by aiding in detecting conditions such as left ventricular dysfunction, CAD, and valvular diseases. Studies have demonstrated AI’s ability to analyze echocardiographic images with accuracy levels comparable to experienced clinicians [121]. This capability enhances the diagnostic process and increases efficiency, particularly in settings where expert cardiologists may be scarce [122]. Moreover, AI’s predictive capabilities have proven beneficial in forecasting adverse cardiac events, such as HF exacerbations and stroke risks. AI-based models can identify high-risk patients early, providing timely interventions and personalized treatment strategies [123]. These predictive models have the potential to substantially reduce hospitalization rates and improve patient outcomes through proactive care [124].

While the promise of AI in cardiology is undeniable, several limitations and challenges hinder its full integration into clinical practice. One of the most significant challenges is the “black box” nature of many AI algorithms, which limits transparency and interpretability. Although AI models demonstrate high diagnostic accuracy, the lack of explanation in their decision-making processes can create trust issues among clinicians and patients [125]. Another critical issue is the quality and diversity of training data. AI models rely on large datasets to make accurate predictions, but many datasets suffer from bias and underrepresentation in cardiology, particularly in diverse or underserved populations. This lack of diversity can lead to models that do not generalize well across different demographic groups, limiting their applicability in real-world settings [120]. Additionally, inconsistent data quality, including noise or artifacts in imaging or diagnostic records, can negatively impact the performance of AI models, leading to inaccurate or unreliable outcomes [124]. Furthermore, the integration of AI into clinical workflows requires significant infrastructural changes. Implementing AI systems in routine clinical practice demands substantial technological, training, and regulatory compliance investments. The integration process is often slow due to regulatory barriers, concerns over patient data privacy, and resistance from healthcare providers who may be apprehensive about replacing traditional methods with automated systems [125].

To address these challenges, several solutions and future directions must be explored. First, enhancing the transparency and interpretability of AI models by developing Explainable AI (XAI) is crucial for increasing trust and acceptance among healthcare professionals [120,123]. XAI provides clinicians with insights into how AI models make decisions, helping them better understand and validate the outputs, which is essential for clinical decision-making. Second, improving the quality and diversity of datasets used to train AI models is critical. This can be achieved through collaborations across institutions, creating more extensive, more representative datasets that cover diverse populations. Additionally, efforts to standardize data collection methods and ensure data consistency across different clinical settings will help improve the reliability and generalizability of AI models [125]. In terms of clinical integration, future research should focus on designing AI systems that work synergistically with clinicians rather than replacing them. AI should be viewed as a tool that enhances clinical capabilities, providing decision support rather than replacing human expertise [126]. Moreover, addressing ethical concerns and ensuring patient data privacy will be crucial for building a regulatory framework that fosters the safe adoption of AI technologies [123].

In the future, AI could also play a significant role in precision medicine. By analyzing vast amounts of patient data, including genomics and clinical records, AI could provide highly personalized treatment plans, ensuring that patients receive the most effective therapies based on their profiles [123]. AI has the potential to significantly advance the field of cardiology by improving diagnostic accuracy, enabling early detection, and enhancing treatment strategies. However, challenges related to transparency, data quality, and clinical integration must be addressed for AI to reach its full potential in clinical practice. By focusing on developing explainable models, improving data diversity, and ensuring ethical practices, AI can become a powerful tool in cardiology, ultimately leading to better patient outcomes.

## Conclusions

A new era in cardiovascular care is emerging with AI integration, enhanced diagnostics, personalized treatments, and patient monitoring. Key advancements, such as automated imaging, predictive models, and wearable technologies, improve clinical decision-making and outcomes. However, challenges like algorithm standardization, data privacy, biases, and implementation costs must be addressed. Collaborative efforts among AI developers, clinicians, and policymakers are essential to ensure ethical deployment, model reliability, and equitable access. As AI evolves, it holds transformative potential to reduce the global burden of CVDs through innovation, precision, and patient-centered care, shaping the future of cardiology.
